# Supplementary material for: Enhanced detection of threat materials by dark-field x-ray imaging combined with deep neural networks
Source: Nat Commun. 2022 Sep 9;13:4651. doi: 10.1038/s41467-022-32402-0 (PMC9463187; doi:10.1038/s41467-022-32402-0)
Supplement: Supplementary file 1 — Supplementary Information [file 41467_2022_32402_MOESM1_ESM.pdf]

Supplementary Information for
"Enhanced detection of threat materials by dark-field x-ray imaging combined with deep neural networks"

T. Partridge[1], A. Astolfo[1,2], F.A. Vittoria[1], M. Endrizzi[1], S.S. Shankar[3], S. Arridge[4], T. Riley-Smith[5], I.G. Haig[2], D. Bate[2,1], A. Olivo[1]

[1]Department of Medical Physics and Biomedical Engineering, UCL, London, WC1E 6BT, UK
[2]Nikon X-Tek Systems Ltd, Tring, Herts, HP23 4JX, UK
[3]Nylers Ltd, Marshall House, Middleton Road, Morden, Surrey, SM4 6RW, UK
[4]Department of Computer Science, UCL, London, WC1E 6BT, UK
[5]XPCI Technology Ltd, The Elms Courtyard, Bromesberrow, Ledbury, HR8 1RZ, UK

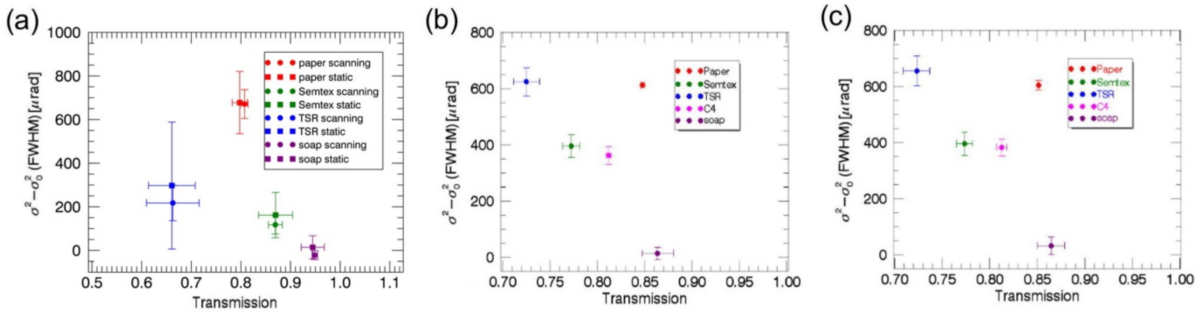

Supplementary Figure 1| comparison between static and scanned acquisitions at various speeds. Panel (a) shows transmission (1 – attenuation) and dark-field values retrieved from a subset of the materials considered in the main paper (soap, paper, Semtex 1A, TSR, marzipan) from both static and scanned acquisitions. In the "static" case, a symmetric pre-sample mask was used, three frames were acquired while displacing the mask each time, and fed to the retrieval algorithm as discussed in reference 15 of the main article. In the dynamic case, acquisitions were performed with an asymmetric masks following the procedure discussed in the methods section. As can be seen, the same values are retrieved for all materials within the experimental uncertainty (one standard deviation), demonstrating that scanned acquisitions do not affect the retrieved values. Panels (b) and (c) show (for a slightly different set of materials, with different thickness) that, at least for the range considered in this experiment, the retrieved values are not affected by the scanning speed, which was 4 times higher for (c) (75 μm/s) than (b) (18.75 μm/s). This is equivalent to stating that (still within the considered range) the retrieved values are not affected by the image resolution, which, along the scanning direction, is determined by how frequently the detector is read out. Error bars correspond to one standard deviation, but note this includes variations inherent to the material.

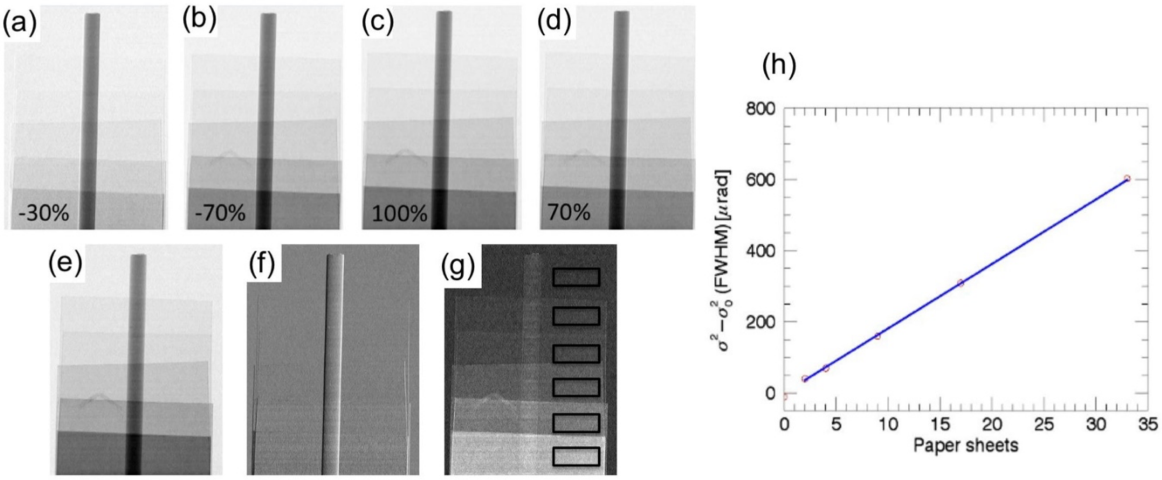

Supplementary Figure 2|Demonstration that scanned acquisition do not affect the linearity of the dark-field signal vs. sample thickness. A simple phantom is used, consisting of a Perspex rod (which causes refraction at the edges) placed over a "step wedge" created with an increasing number of paper sheets. Panels (a), (b), (c) and (d) show the raw images collected at the four IC points as determined by the relative shifts in the period of the asymmetric mask (see Fig. 7 in the main article). These are then fed to the retrieval algorithm to produce the attenuation (e), refraction (f) and dark-field (g) images. Regions of interest are then isolated in all areas of the dark-field image corresponding to the different paper thicknesses (black rectangles in (g)). Average pixel contents from each region of interest are then plotted against the corresponding number of paper sheets (proxy for paper thickness), obtaining a linear relation (h).

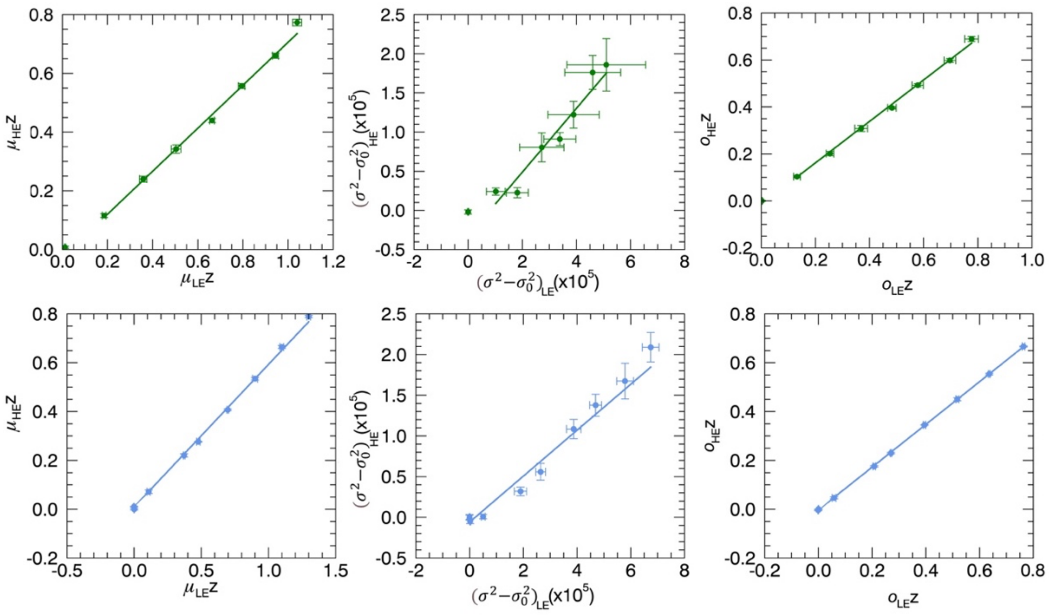

Supplementary Figure 3| thickness-independent signal ratios. Demonstration (on the same example materials used in Fig. 2(c) in the main article) that the ratios chosen for the 3D separation of threat vs. benign materials shown in Fig 2(d), namely ratios of $\sigma^2 - \sigma_0^2$, *t* and *o* at high and low energies, allow obtaining thickness-independent quantities. The above plots show high energy (HE subscript in the graphs) vs. low energy (LE subscript) values of *t*, $\sigma^2 - \sigma_0^2$ and *o* (left to right) for eight different thicknesses of Semtex H (top row) and soap (bottom row). The linear relation observed in all cases demonstrates that the division of the two corresponding quantities reported in each graph is constant vs. thickness. Error bars correspond to one standard deviation.

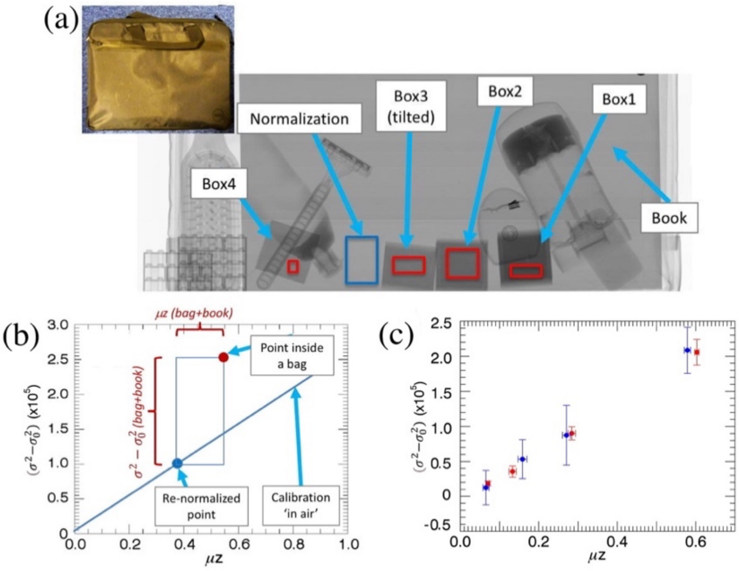

Supplementary Figure 4| Renormalisation of signals from targets buried underneath layers of different materials. A series of boxes containing different thicknesses of Semtex were placed in a padded bag containing a thick book and other cluttering objects (panel (a); a photo of the bag is shown in the inset). A "renormalisation" area is identified, where only the overlapping materials (bag and book) are present (blue rectangle in (a)). This allows calculating the additional signal as shown in (b), subtracting which "renormalises" the Semtex signal. Panel (c) shows that, post-renormalisation, values extracted from different Semtex thicknesses inside (blue points) and outside (red points) the bag lead to the same outcome. Error bars correspond to one standard deviation.

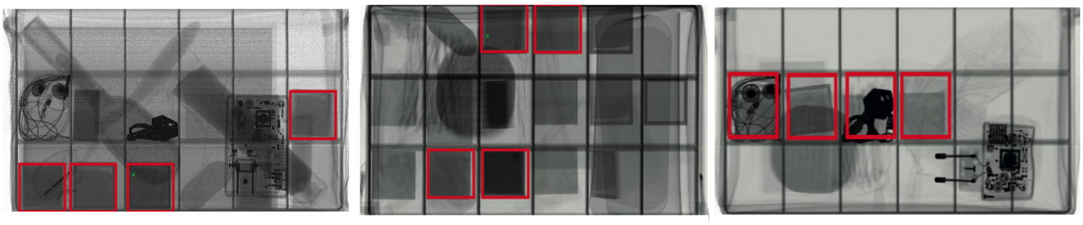

Supplementary Figure 5| Example images from the PoC test 1 dataset. Red boxes indicate the positions of concealed threat materials. It can be seen how, to the human eye, separating threat and non-threat materials may not be easy, supporting a CNN-based approach which makes them more easily separable by representing the data onto a higher-dimensional space.

Trial	True Positives	False Positives
GoogleNet (without dark-field signal)	82.1%	29.2%
GoogleNet (with dark-field signal)	87.3%	21.4%
Inception ResNet (without dark-field signal)	79.7%	30.1%
Inception ResNet (with dark-field signal)	84.2%	23.2%

Supplementary Table 1|Results obtained by processing the dataset of PoC test 2 with standard CNN architectures (GoogleNet and Inception ResNet). The CNNs were trained with the softmax loss (as it showed consistent convergence over this problem), with the other training details similar to those provided in the methods section. This exercise allows to appreciate the improved performance obtained with our "split network" architecture (compare the above results with those of table 1 in the main article), and that the inclusion of the dark-field signal improves the overall performance also when more standard CNN architectures are used.

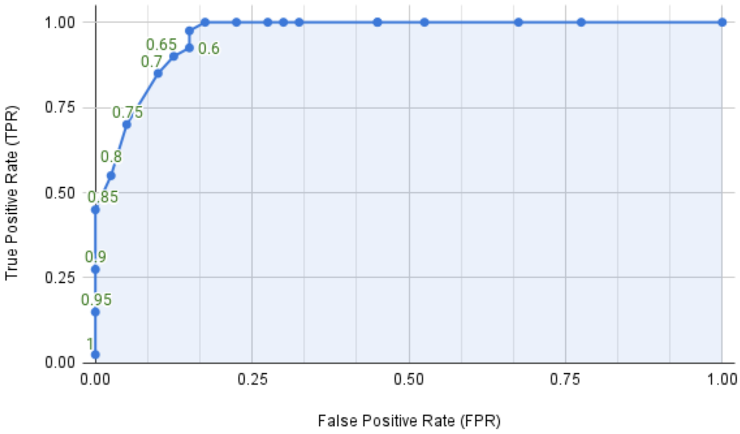

Supplementary Figure 6| Receiver Operating Characteristic (ROC) curve for the dataset of PoC test 2. This was obtained while varying the positive (i.e. "explosive") classification threshold, for the split network CNN with the dark-field signal included (result reported in the second row of Table 1 in the main article). The threshold decreases monotonically from 1 (bottom left of the graph) to 0 (top right) in steps of 0.05; some thresholds are reported in the graph, but not all, to avoid clutter. As summarised in table 1 in the main article, a true positive rate of 100% is obtained with a threshold of 0.5, at which the false positive rate is around 17.5%.

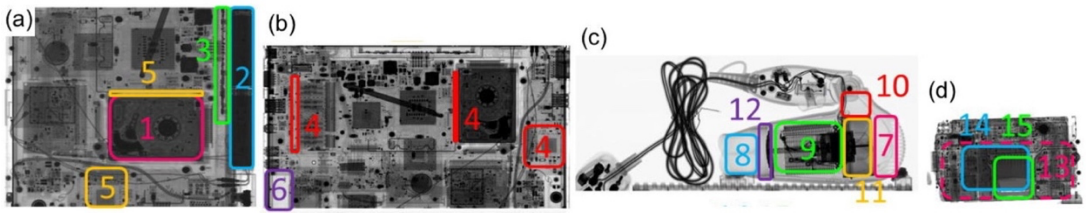

Supplementary Figure 7| Areas of concealment of C4 in electrical items. These are shown for laptop (a, b), hair drier (c) and mobile phone (d), with numbered boxes showing the various areas of concealment.

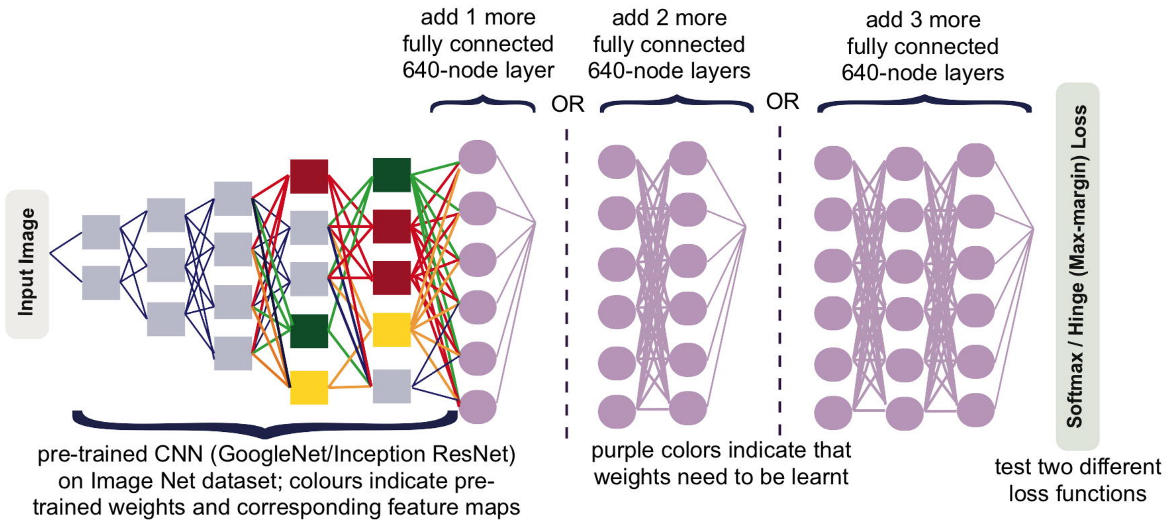

Supplementary Figure 8| Schematic of our Type I architecture. A CNN pre-trained on ImageNet is augmented with 1, 2 or 3 additional fully connected layers for performing transfer learning over our small dataset. The grey feature maps (rectangles) in the network indicate pre-trained weights, while the red, green and yellow ones show that some of the pre-trained feature maps are altered during the fine-tuning process. We either use a softmax or a hinge loss for training. We tested both GoogleNet and Inception ResNet as CNN architectures.

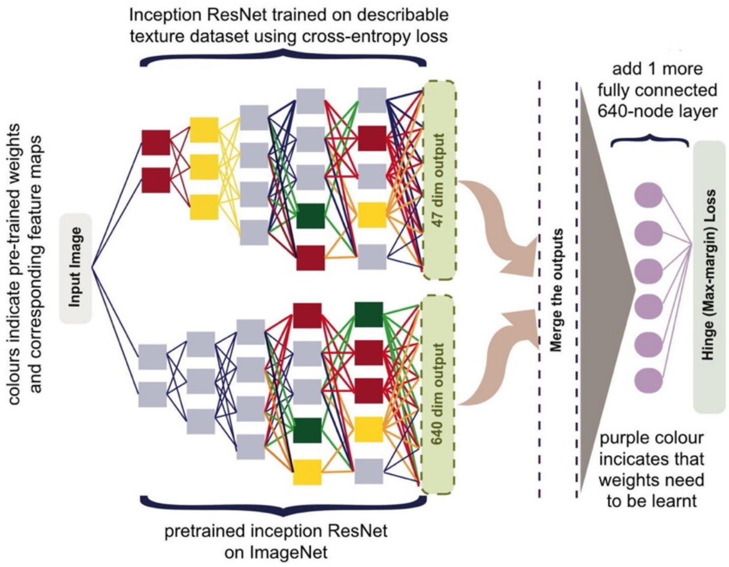

Supplementary Figure 9| Schematic of our Type II architecture. We fuse the output of an Inception ResNet trained for texture recognition with the 640-dimensional last layer output of another Inception ResNet, and train this merged architecture with hinge loss for classifying explosives and non-explosives.

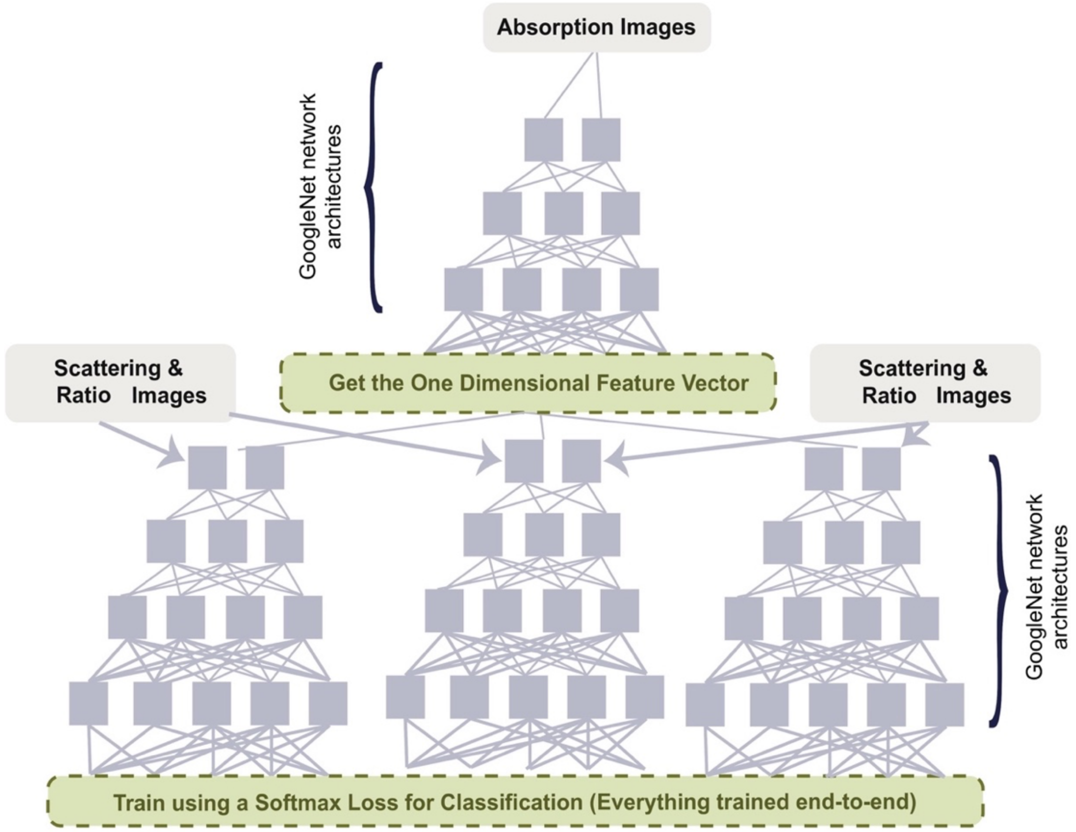

Supplementary Figure 10| Split-network CNN Architecture. The first network only takes absorption images as input, as these were proven to be sufficient to segregate the electrical items. This network's feature vectors are then fed into three different networks, along with scattering (dark-field) and ratio images. The network is trained end-to-end with a softmax loss function. We empirically found that the absorption images help to segment into object parts; then around those, we learn the texture information of the explosive with dark-field and ratio images. Having three different networks in the bottom layer was an empirical choice, which gave the best results.
